# Changes in Stream Peak Flow and Regulation in Naoli River Watershed as a Result of Wetland Loss

**DOI:** 10.1155/2014/209547

**Published:** 2014-07-07

**Authors:** Yunlong Yao, Lei Wang, Xianguo Lv, Hongxian Yu, Guofu Li

**Affiliations:** ^1^College of Wildlife Resource, Northeast Forestry University, Harbin 150040, China; ^2^Key Laboratory of Wetland Ecology and Environment, Northeast Institute of Geography and Agroecology, CAS, Changchun, Jilin 130012, China; ^3^College of Architecture and Civil Engineering, Heilongjiang Institute of Science and Technology, Harbin 150027, China

## Abstract

Hydrology helps determine the character of wetlands; wetlands, in turn, regulate water flow, which influences regional hydrology. To understand these dynamics, we studied the Naoli basin where, from 1954 to 2005, intensive marshland cultivation took place, and the watershed's wetland area declined from 94.4 × 10^4^ ha to 17.8 × 10^4^ ha. More than 80% of the wetland area loss was due to conversion to farmland, especially from 1976 to 1986. The processes of transforming wetlands to cultivated land in the whole Naoli basin and subbasins can be described using a first order exponential decay model. To quantify the effects of wetlands cultivation, we analyzed daily rainfall and streamflow data measured from 1955 to 2005 at two stations (Baoqing Station and Caizuizi Station). We defined a streamflow regulation index (SRI) and applied a Mann-Kendall-Sneyers test to further analyze the data. As the wetland area decreased, the peak streamflow at the Caizuizi station increased, and less precipitation generated heavier peak flows, as the runoff was faster than before. The SRI from 1959 to 2005 showed an increasing trend; the SRI rate of increase was 0.05/10*a*, demonstrating that the watershed's regulation of streamflow regulation was declined as the wetlands disappeared.

## 1. Introduction

Wetlands are a declining resource that occupy less than 9% of the earth's land area; however, their value and contribution to humanity and the ecosystem are far more valuable than their small area implies [1, 2]. Wetlands provide such ecosystem services as improved water quality, provision of flood control, mitigation of climate change, and groundwater recharge [3–7]. Concerns about the reduced area and condition of many of the world's wetland systems are growing, as more and more wetlands are converted to agricultural land or lost to urbanization [3, 8].

Wetlands in catchment areas play an important role in regional hydrological processes. While hydrology determines the character of wetlands, wetlands also influence regional water flow regimes [4]. Inland wetlands are known to provide a wide array of hydrological services, but many of the services are not well understood [9]. Understanding how different types of wetlands, as well as their gain or loss, contribute to a watershed's flood mitigation function is a large knowledge gap for policy and decision makers [2].

A basic question remains: what proportion of a given watershed area should be represented by wetlands to achieve flood control? Opinions differ on the advantages and disadvantages of preserving and restoring wetlands in a watershed's upper reaches [1], but floodplains are known to be critical in mitigating flood damage, as they store large quantities of water and effectively reduce the height of flood peaks and downstream flooding risk. Hey and Philippi (1995) suggested that restoring approximately 13 million acres (5.3 million ha) of wetlands in the Upper Mississippi and Missouri Basins would have provided enough floodwater storage (about 1 m deep) to accommodate the excess river flow from the disastrous flood in Midwestern USA in 1993 [10]. If those 5.3 million hectares were added to the existing 7.7 million hectares in the region, an estimated 7% of the watershed would be sufficient to address even extreme event floods on a large scale. Mitsch and Gosselink suggested that a range of 3–7% of temperate-zone watersheds should be occupied by wetlands to provide adequate flood control and water quality values for the landscape [1].

Sanjiang Plain, located in the northeast of Heilongjiang Province, Northeast China, previously contained the largest continuous area of freshwater wetlands in China—it was in a completely natural and untouched condition before the 1950s. From the late 1950s to the early 1990s, however, a large number of farms were established across the plain, leading to the loss of wetlands (nearly 80% of the freshwater wetlands in Sanjiang Plain have been transitioned to other uses) and a decline in the condition of the remaining wetlands due to the changes in hydrology.

At a regional scale, the ecosystem services provided by the wetlands remaining have declined dramatically. The total annual ecosystem service values in Sanjiang Plain have declined by about 40% between 1980 and 2000. This substantial decline is largely attributed to the loss of wetlands [11]. Due to limited data available from Northeast China, few reports are available to describe the impacts of wetland loss on the peak flow of Naoli River Basin as the wetlands have been converted to cropland. The whole water storage capability of wetland of Sanjiang Plain was originally 17.15 × 10^8^ m^3^ in 1950s [12]. This storage capability declined with the cultivation of wetland areas, which has most likely produced heavier peak flows during extreme rainfall events. The annual runoff declined dramatically since 1950s, and the hydrological regime has also changed [13–18]. However, the impact of wetland cultivation on the watershed's peak flow and regulation has not yet been quantified.

This study focused on the effect of wetland cultivation on the peak flow and regulation function of the Naoli River catchment. The study goal was to assess whether the hydrological function of wetlands, especially the flood control function, has declined as a result of wetland loss over the last 50 years.

## 2. Study Area

**Figure 1:**
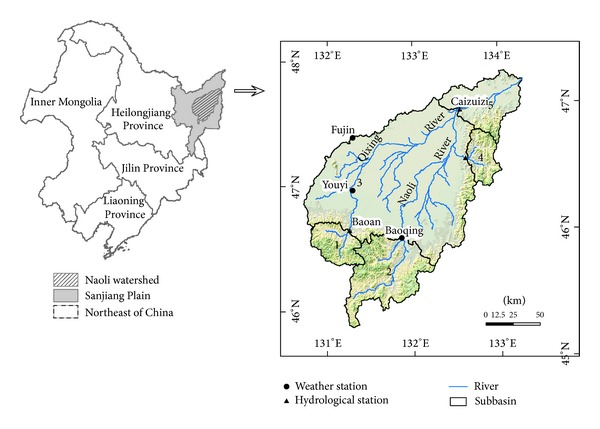
The location sketch of study area.

The Naoli River watershed (131° 31′–134° 10′E and 45°43′–47° 45′N) is located within the Sanjiang Plain in Heilongjiang Province (Figure 1), covering 2.42 × 10^4^ km^2^. It is estimated that mountains occupy 38.3% of the total area; the plain occupies 61.7%. The Naoli River, the primary tributary of the Wusuli River, originates from the Qliga Mountain of the Wanda Mountain in Boli County, Heilongjiang Province, and flows into the Wusuli River in the Dong'an Town of Raohe County. The river's overall length is 283 km. The Naoli River watershed lies in a temperate zone with a continental monsoon climate. The mean annual temperature is 1.6°C, with an average temperature of −21.6°C in January and 21.4°C in July. The mean annual precipitation is 565 mm, while mean annual evaporation is 542.4 mm. The terrain in the Naoli River watershed is flat and low, with an average altitude of about 60 m. The landscape is characterized by an extensive river floodplain, with widely distributed dish-shaped swales and limited surface runoff. A predominantly clay substrate hampers surface water infiltration, which originally led to a formation of extensive wetlands (including different kinds of freshwater marshland, riverine wetland, and ponds), originally taking up one-fourth (or 94.4 × 10^4^ ha) of the Sanjiang Plain. Unique natural and climatic conditions once created a rich area of wetlands and a unique ecological environment. In particular in the vast plain region, which represents a broad river floodplain, different kinds of wetlands were formed. Because of subsequent agricultural activities, however, the plain's total wetland area in 2000 was 3460.0 km^2^, which was only 36.7% of the original area of 9440.0 km^2^ in 1954. Nearly 80% of the area was turned into crop's land during this time. As a result, the structure and function of the watershed have changed dramatically.

Based on the location of the hydrological stations, for study purposes, we divided the basin into five subbasins, marked by Arabic numerals as shown in Figure 1. There were two subbasins in the headwaters, two subbasins in transfer zone, and one in the depositional zone.

## 3. Data and Method

### 3.1. Data Source

There are only four hydrological stations in the Naoli River watershed: Baoqing, Bao'an, Caizuizi and Hongqiling Stations (see Figure 1). Baoqing and Bao'an Stations are located in the upper reaches of Naoli River, and Caizuizi and Hongqiling are located in the middle reaches. Data from Bao'an Station were insufficient and could not be used. Data from Hongqiling Station were also not appropriate for this study, due to a shorter observation period and smaller catchment area. As such, for this study, runoff data were used from Baoqing Station (catchment area of 3689 km^2^) and the Caizuizi Station (catchment area of 20796 km^2^). Daily measured runoff data from 1956 to 2005 were used from Baoqing Station and Caizuizi Station in Naoli River watershed, from which we calculated monthly and annual mean runoff.

Daily precipitation data from 1956 to 2005 were used from weather stations in Baoqing County, Youyi County, and Fujin City, provided by the Heilongjiang Sharing Service Center of Weather Scientific Data. Precipitation data from Baoqing Station include the annual mean precipitation measured in the weather station of Baoqing County from 1956 to 2005. Additionally, precipitation from Caizuizi Station is represented by the average of the annual mean precipitation measured in the Baoqing, Youyi, and Fujin weather stations. Sources of the hydrological data and precipitation can be found in Table 1.

The land use datasets of the watershed were obtained during relatively cloud-free days in September 1980 (MSS data), August 1996 (TM data), September 2000 (TM data), and September 2005 (TM data). Land use data from the 1950s was derived using a 1950's topographic map. All the land use datasets were provided by the China Wetland Scientific Database.

### 3.2. Method

#### 3.2.1. Wetland Change Detection

The land use datasets provided by the China Wetland Scientific Database were already classified into seven land use/cover categories: woodland, grassland, farmland, water body, wetland, residential land, and barren land. For the purposes of our research, we regrouped the classification into two groups: wetlands and nonwetlands. The total wetland areas during the different periods were calculated using ARC/INFO (ESRI, 1994) Geographical Information System (GIS) software; the wetland areas of the whole basin and subbasins were also compared during the different periods.

#### 3.2.2. Mann-Kendall-Sneyers Test

Many time-series analyses methods are used by different researchers [19–22]. In this study, The Mann-Kendall-Sneyers test had the greatest ability to test the trend of hydrological time series data [23–25]. As such, the test was used to analyze the phase features of the annual mean runoff records from the Baoqing hydrological station and the Caizuizi hydrological station in the Naoli River watershed.

Let *x*_1_, *x*_2_,…, *x*_*n*_ be the time series data points. For each element *x*_*i*_, we computed the numbers *m*_*i*_ of elements  *x*_*j*_ preceding it (*j* < *i*), such that *x*_*j*_ < *x*_*i*_. Under the null hypothesis (no trend), the test statistic

(1) $$\begin{array}{c} t_{k}=\overset{k}{\underset{i=1}{\sum }}mi\;\left(2\leq k\leq n\right) \end{array}$$

is normally distributed with mean and variance given by the following equations:

(2) $$\begin{array}{c} \overset{-}{t_{k}}=E\left(t_{k}\right)=\frac{k\left(k-1\right)}{4},\\ \mathrm{Var}\left(t_{k}\right)=\frac{k\left(k-1\right)\left(2k+5\right)}{72}. \end{array}$$

Let

(3) $$\begin{array}{c} u_{k}=\frac{t_{k}-\overset{-}{t_{k}}}{\sqrt{\text{var}\left(t_{k}\right)}} \end{array}$$

be the normalized variable, which is the forward sequence. The backward sequence *u*_k_* is calculated using the same equation, but with a reversed series of data.

When the null hypothesis is rejected (i.e., if any of the points in the forward sequence are outside the confidence interval), it indicates an increasing (*u*_*k*_ > 0) or a decreasing (*u*_*k*_ < 0) trend. The sequential version of the test enables detection of the approximate time of trend occurrence by locating the intersection of the forward and backward curves of the test statistic. If the intersection occurs within the confidence interval, then it indicates a change point [26, 27].

#### 3.2.3. Definition of Streamflow Regulation Index

According to the hydrological theory, the runoff is tightly correlated to the precipitation under the nature condition. When the precipitation varies, the runoff will correspond. To quantify the impacts of wetland loss on the streamflow regulation, we defined the streamflow regulation index through the following formula:

(4)  SRI=RcvPcv While,  Rcv=Rsd⁡Ravg,  Pcv=Psd⁡Pavg,

where SRI was the streamflow regulation index, *R*_cv_ was the coefficient of variation of runoff, *R*_*sd*⁡_ was the standard deviation of runoff, and *R*_avg_ was the average of the runoff; *P*_cv_ was the coefficient of variation of precipitation, *P*_*sd*⁡_ was the standard deviation of precipitation, and *R*_avg_ was the average of the precipitation. SRI can be interpreted as follows: when the variation coefficient of precipitation increased, but the variation coefficient of runoff was not equally increased, then the streamflow regulation index became smaller, so the ability of the streamflow regulation of the watershed was increased; otherwise, it was decreased. The daily runoff and precipitation of Caizuizi Station was used from June to November from 1959 to 2005.

## 4. Results 

### 4.1. The Process of Wetland Cultivation in Subbasins

**Figure 2:**
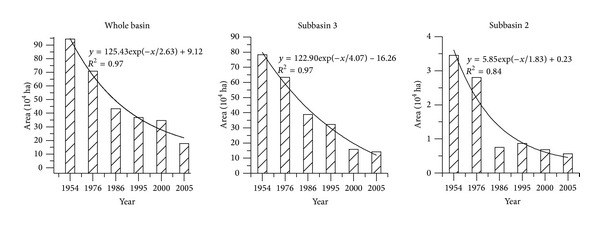
Wetlands cultivation in the whole basin, subbasin 2, and subbasin 3.

The area of wetlands in each subbasin was different in 1954 Table 2. The area of wetlands in subbasins 3 and 5 was higher than other subbasins (1, 2, 4); the percentage, respectively, was 51.8% and 42.5%. Individual subbasin areas are shown in Figure 2. There were no hydrological observations in subbasin 5. The total area of subbasins 2 and 3 is 18,801.3 km^2^, accounting for nearly 80% of whole catchment. So, to compare wetland loss impacts on hydrological characteristics, wetland cultivation of the whole catchment and subbasins 2 and 3 was analyzed separately.

The loss of wetlands across the full Naoli watershed was dramatic between the 1950s and 2005. The wetland area declined from 94.4 × 10^4^ ha to 17.8 × 10^4^ ha; more than 80% of the wetlands were lost. The loss rate between 1976 and 1986 was more rapid than during other years. The dynamics in the two subbasins included in the study were almost the same: the area of wetland declined from 2.8 × 10^4^ ha and 63.4 × 10^4^ ha to 0.8 × 10^4^ ha and 38.8.9 × 10^4^ ha in subbasins 2 and 3, respectively; about 80% of the wetland was converted to farmland. The percentage of wetland declined to 10.5% in subbasin 3. After 1995, the area of wetland declined less rapidly and wetland cultivation nearly stopped.

The process of wetland loss in the full basin and subbasins can be described using first order exponential decay model; the fitting degree was high (whole basin: *R* ^2^ = 0.97, subbasin 2: *R* ^2^ = 0.84, and subbasin 3: *R* ^2^ = 0.97). The rapid decrease in the number and areas of marshes was largely attributed to extensive agricultural reclamation under the “Food First” agricultural policy. This resulted in many negative ecological consequences, such as extreme peak flow and habitat loss.

### 4.2. Changes in the Peak Flow 

#### 4.2.1. Variation of Annual Maximum Peak Flow

The annual maximum peak flow was derived from the daily runoff data collected between June and November from 1959 to 2005. The maximum flow of a day in a year is set as the annual maximum peak flow of that year. Based on the annual precipitation, we divided the 47 study years into three types of hydrological years: wet years, dry years, and normal years. If the annual precipitation of one year was 10 percent greater than the average annual precipitation, this year was defined as a wet year; if annual precipitation was 10 percent less than the average annual precipitation, this year was defined as a dry year. The rest of the years, the years where rainfall was within 10% above or below average, were classified as normal years.

**Figure 3:**
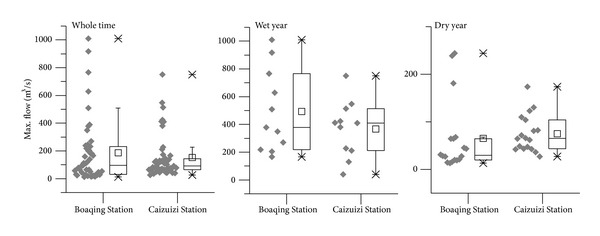
Maximum peak flow of Baoqing Station and Caizuizi Station from June to November between 1959 and 2005.

The variation of these types of years is shown in Figure 3. During the wet years, the peak flows in Baoqing Station (upstream) were greater than those in Caizuizi Station (midstream) in most of the years. The situation was opposite during the dry year. As a whole, there were 22 years when peak flows in Baoqing Station were greater than those in Caizuizi Station. The maximum peak flow of 1 010 m^3^/s in Baoqing Station happened in 1964 between 1959 and 2005, while the peak flow was only 547 m^3^/s in Caizuizi Station, which is a lower value by 46%. Because of the wetland cultivation, the peak flow of Caizuizi Station became greater. The maximum peak flow in Caizuizi Station occurred in 1981 at 750 m^3^/s, while the flow in Baoqing Station was 629 m^3^/s. The watershed's regulation function of peak flow declined as the wetland was lost.

**Figure 4:**
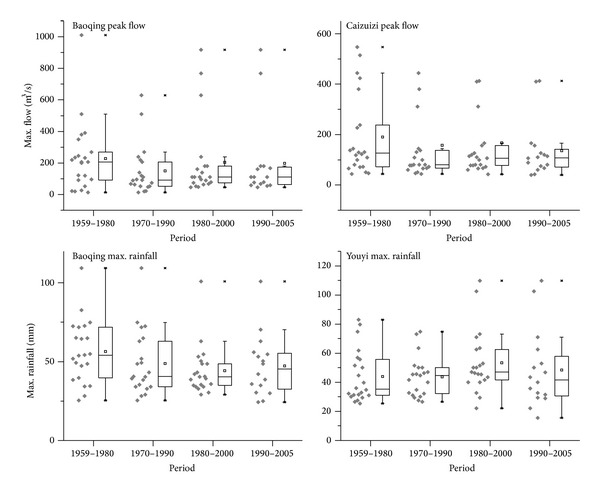
Peak flow and maximum rainfall magnitude across four time periods. Boxplots represent data from different periods; the horizontal line in each boxplot is the median, while the square box holds the mean.

Using time series of annual maximum peak flows from Baoqing Station and Caizuizi Station, we also compared average maximum peak flow from four overlapping 20 yr periods (1959–1970 (hereafter called the 1960s); 1970–1990 (1970s); 1980–2000 (1980s); and 1990–2010 (1990s)) (see Figure 4). In general, maximum flow events were largest in 1960s and then declined in 1970s, increased in 1980s, and decreased in 1990s. These variations can be attributed to the difference of weather patterns and wetland loss. However, the comparison of average maximum rainfall from Baoqing Station and Youyi Station (representing the precipitation regime of Caizuizi subbasin) showed no such variation (see Figure 4).

#### 4.2.2. Different Runoff Process at Same Precipitation Regime

**Figure 5:**
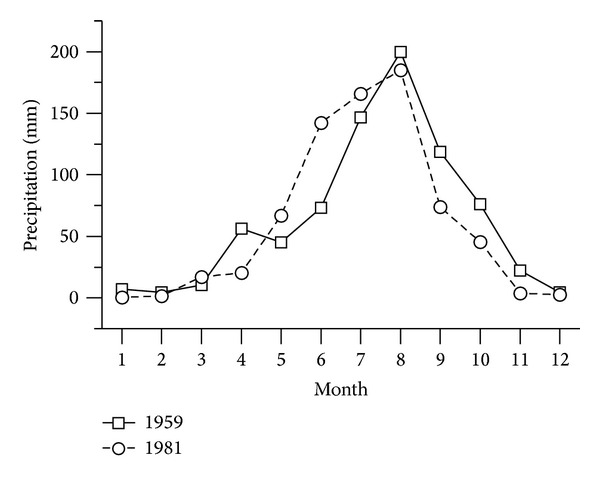
Annual precipitation distribution in Caizuizi Station in 1951 and 1981.

The rainfall regime was almost the same in 1959 and 1981. The annual precipitation was a little greater in 1959 than that in 1981, respectively (765 mm versus 726 mm). The monthly precipitation is shown in Figure 5; it is clearly almost the same. However, the runoff process was different, especially in terms of peak flow.

**Figure 6:**
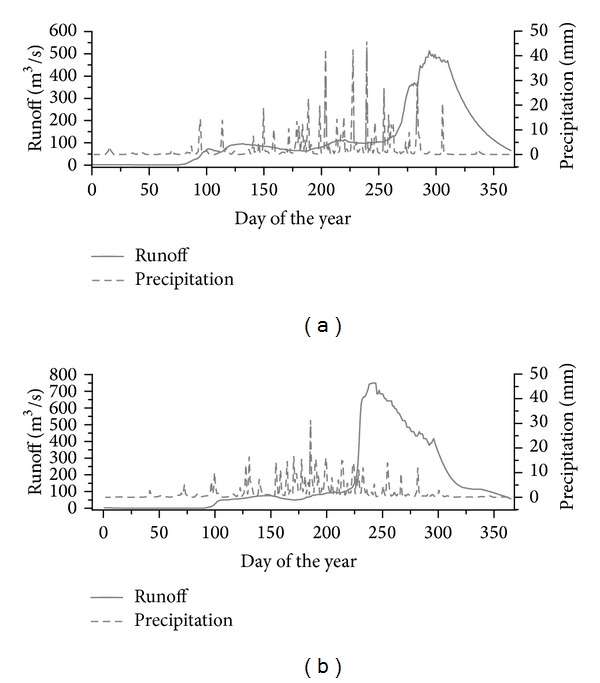
Daily rainfall and runoff in Caizuizi Station (a) 1959, (b) 1981.

Daily precipitation and runoff details are summarized in Figure 6; the results are summarized in the following three points. First, the maximum peak flow was different between 1959 and 1981; the difference was an increase of nearly 50%. The maximum peak flow was 514 m^3^/s in 1959; it was 750 m^3^/s in 1981. Second, the rise-time from daily mean runoff to the peak flow was different. The rise-time was 36 days in 1959; it became shorter in 1981, falling to about 18 days. The time from the peak flow to daily mean runoff was also different. It was longer in 1981 than that in 1959, at 73 days and 54 days, respectively. This demonstrates that as more water flowed out of watershed, the wetland's storage capability dramatically declined. Third, the total amount of precipitation experienced when runoff reached its maximum was also different. The precipitation was 737 mm in 1959, representing 96.3% of total precipitation of this year. The precipitation was 632 mm in 1981, representing 85.5% of total precipitation. Less precipitation could generate heavier peak flow.

### 4.3. Changes in Streamflow Regulation

**Figure 7:**
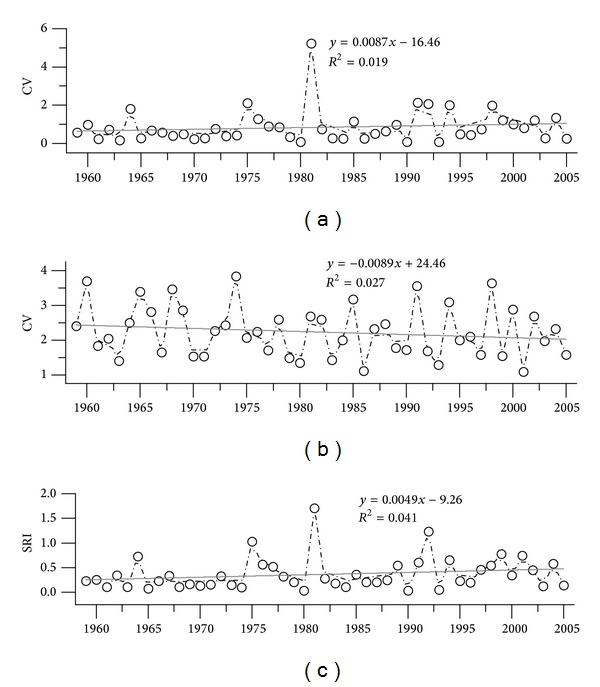
Variation coefficient of runoff, rainfall, and SRI in Caizuizi Station: (a) runoff, (b) rainfall, (c) SRI.

In 1954, most of the area (51.8%) in subbasin 3 was still covered by wetlands; these wetlands were subsequently removed and were degraded more rapidly than those in other subbasins. As such, we only analyzed the streamflow regulation index of subbasin 3 (Caizuizi Station) in this section. The variation coefficient of runoff and rainfall is provided in Figure 7. The variation coefficient of runoff showed an ascending trend; the rainfall was opposite, with a descending trend. However, results of Mann-Kendall-Sneyers test showed that the ascending trend of variation coefficient of runoff was not obvious (*Z *= 1.16, *a*_1_ = 0.12 > 0.05); the descending trend of coefficient of variation of rainfall was also not statistically significant (*Z *= −1.08, *a*_1_ = 0.14 > 0.05). When the impacts of rainfall were eliminated, the streamflow regulation index had an obviously ascending trend (Figure 7).

The results of Mann-Kendall-Sneyers test showed an ascending trend in the streamflow regulation index (*Z *= 1.72, *a*_1_ = 0.04 < 0.05). Using linear regression, we found that the streamflow regulation growth rate index was 0.05/10*a*. Despite many water conservancy projects in this watershed, the streamflow regulation function of watershed still declined.

These results showed an increase in the runoff coefficient of variation over time, while the peak flow was decreasing. This seems contradictory; however, the data analysis suggests three main reasons for this. (1) The runoff variation coefficient showed an ascending trend. However, the Mann-Kendall-Sneyers test revealed that the ascending trend of variation coefficient of runoff was not significant. (2) In order to control flood and drought, many water conservancy projects were built, including more than 10 reservoirs. Longtouqiao Reservoir, built in 2003, is the largest reservoir in Sanjiang Plain and plays an important role in flood prevention. (3) As discussed previously, the lack of data availability and quality presents study limitations. The previously discussed studies of peak flow responses have relied mainly on statistical methods and therefore have been limited by the availability of the data. There is only one hydrological station in the middle of the river; as such, some of the detailed hydrological changes experienced during wetland cultivation may be missed.

## 5. Discussion

### 5.1. The Contribution of Wetland Transformation

**Figure 8:**
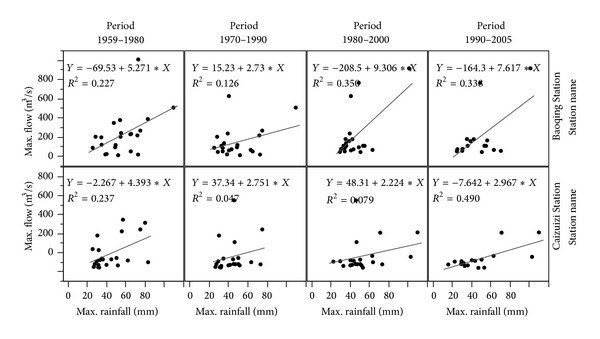
The relationships between maximum flow and maximum rainfall in different period.

From the previous section, we know that the maximum rainfall does not vary significantly between the different time periods. However, during the time of wetland loss, the relationships between maximum flow and the maximum rainfall become more tightly connected. The linear regression coefficient (*R* ^2^) increases, especially from the 1970s to 1990s (see Figure 8), and the* R* ^2^ increased from 0.12/0.04 to 0.33/0.49 in Baoqing Station/Caizuizi Station. This means that the runoff increases in response to the rainfall as the wetland loss increases.

Following the conversion of wetlands to croplands, Sanjiang Plain became an important commodity grain production base in China [28, 29]. The area of farmland in Naoli watershed accounts for one-third of the total farmland of Sanjiang Plain. As such, the impacts of flood damage must be considered, as that damage directly impacts national food security.

### 5.2. The Impact of Wetlands Loss on the SRI

**Figure 9:**
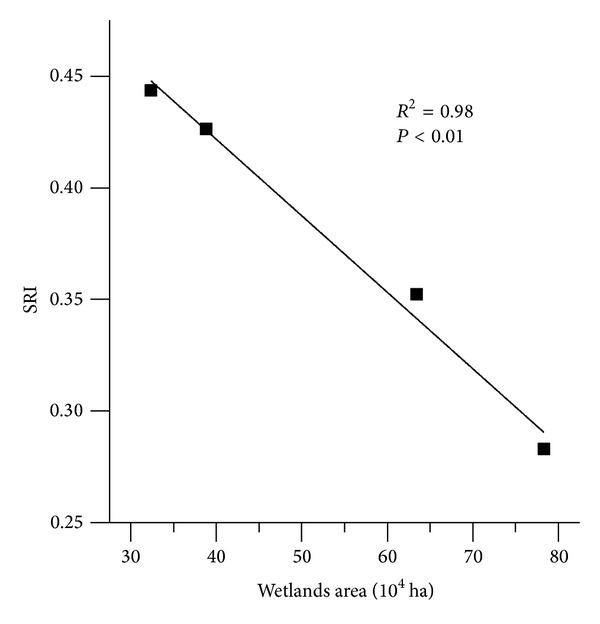
Scatter plot of wetlands area in subbasin 3 and the SRI of different 20 yr periods.

To clearly understand the relationships between wetlands loss and SRI, we used SRI time series from Caizuizi Station to calculate the average SRI from the four overlapping 20 yr periods described above. The scatter plot of the wetland area of subbasin 3 against the SRI shows a negative relationship between the wetland area and SRI (Figure 9). The stream regulation function decreased as the wetlands were lost from subbasin 3.

It is widely recognized that wetlands provide important hydrologic functions in a watershed [30–32]. While the importance of individual wetlands for mitigating flood intensity and duration is understood, the degree to which wetland development affects flooding at the watershed or ecosystem level is rare. From the literature review, we found some similar results. Brody et al. (2007) examined the relationship between wetland alteration and coastal watershed flooding in Texas and Florida over a 12-year period and found that specific types of federal permits exacerbate flooding events [33]. Using the HEW concept in SWAT to assess the effects of wetland restoration for a 4506 km^2^ in Minnesota, Wang et al. (2010) found that a reduction of approximately 10–20% of the wetlands in the study area resulted in a considerable increase in peak discharge [34]. Our research result is another evidence to prove the hydrological service of wetland in a watershed.

Because of the limitation of the statistic methods, we could not quantify the impacts of wetland loss on the hydrological process. In the future, we recommend using distributed hydrological model, such as the Soil and Water Assessment Tool (SWAT) [35], to analyze the impacts of wetland loss/restoration on the hydrological process. But lack of hydrological and meteorological data is a major challenge for using hydrological model in this region of China.

## 6. Conclusion

This study used the Naoli River watershed to study the process of wetland loss and the impact of that loss on the peak streamflow and regulation. Study findings provide a scientific foundation that may help inform local water resources management. Key findings were as follows.

1. Wetlands in the study area declined from 94.4 × 10^4^ ha to 17.8 × 10^4^ ha between 1950s and 2005, reflecting a loss of approximately 80%; the loss rate was most rapid from 1976 to 1986.
2. Over the period of wetland loss, the peak flow at Caizuizi Station increased, particularly during dry years; lower precipitation generated heavier peak flows, and runoff after rainfall events increased. Streamflow regulation declined with the decrease in wetland area; the SRI growth rate was calculated as 0.05/10*a* and was tightly related to wetland loss. Watershed manager must remain attentive to the rapid impacts of extreme rainfall events in the future.

## Figures and Tables

**Table 1:** Data sources on daily precipitation, streamflow, and land use.

Data type	Station name	Year	Data provider
Precipitation	Baoqing	1956 to 2005	Heilongjiang Sharing Service Center of Weather Scientific Data
	Youyi	1952 to 2005	
	Fujin	1961 to 2005	

Runoff	Baoqing	1955 to 2005	Department of Hydrology of Heilongjiang Province
	Baoan	1957 to 2005	
	Caizuizi	1956 to 2005	
	Hongqiling	1971 to 2005	

Land use	—	1950s, 1980, 1996, 2000, 2005	China Wetland Scientific Database

**Table 2:** Percentage of wetlands in each subbasin in 1954.

Subbasin name	Subbasin number	Area of subbasin (km^2^)	Percentage of wetlands (%)
Bao'an	1	1354.2	6.6%
Baoqing	2	3684.2	9.4%
Caizuizi	3	15117.1	51.8%
Hongqiling	4	1102.8	8.7%

Xiayou	5	2553.6	42.5%
